# Associations between dietary habits and depressive disorder: A diet-wide Mendelian randomization study

**DOI:** 10.1097/MD.0000000000047516

**Published:** 2026-02-20

**Authors:** Meng Li, Kun Wang, Jianmin Liu, Juan Jia, Hai Yuan, Derong Kong, Wenfeng Li

**Affiliations:** a Intensive Psychiatry, Zhengzhou Eighth People’s Hospital, Zhengzhou, Henan Province, China; b Department of Neurology, Beijing Puren Hospital, Beijing, China.

**Keywords:** associations, depressive disorder, dietary habits, Mendelian randomization study

## Abstract

Dietary habits are associated with the onset and progression of major depressive disorder (MDD). Nevertheless, a direct association between diet and MDD remains unclear. Therefore, we used a two-sample Mendelian randomization (MR) method to systematically investigate causal relationships between dietary habits and MDD. We collected summary statistics for dietary habits based on publicly available genome-wide association data from the UK Biobank (n = 461,981) and summary statistics of data from the Psychiatric Genomics Consortium (n = 484,598). MR estimates from the genetic tools were combined using a weighted median method. To validate the robustness of the results, we compared the results of the weighted median with those of the inverse variance weighting, weighted mode, and MR-PRESSO. The results MDD was associated with 5 types of dietary habits, including salt added to food (odds ratio (OR): 1.0182, 95% confidence interval (CI): 0.0087–0.0273, *P* = .0001), beef intake (OR: 0.9395, 95% CI: −0.0964 to −0.0284, *P* = .0003), oily fish intake (OR: 1.0592, 95% CI: 0.0084–0.1065, *P* = .0252), bread intake (OR: 0.9313, 95% CI: −0.1366 to −0.0057, *P* = .0420), and alcohol intake frequency (OR: 1.0085, 95% CI: 0.0006–0.0163, *P* = .0341). The results suggest a causal relationship between dietary habits and MDD, identifying potential protective and risk factors. These results provide a new reference for the prevention of MDD via dietary regulation.

## 1. Introduction

The World Health Organization reports that approximately 280 million people worldwide suffer from major depressive disorder (MDD), making it the leading cause of mental health disabilities.^[1]^ Due to the impact of the coronavirus disease (COVID-19) pandemic, the prevalence and annual incidence of MDD have continued to rise, and the number of MDD patients worldwide has increased by 27.6% during the pandemic.^[2]^ MDD is thought to be caused by an interaction between genetic and environmental factors, with genetic factors estimated to account for approximately 37% of MDD cases.^[3]^ A considerable number of patients can cannot obtain relief from current antidepressants and have poor compliance with antidepressants due to side effects. It is compelling to seek new prevention and treatment strategies in addition to drugs.^[4]^

Human health is significantly affected by individual dietary habits and decisions. Neurological processes associated with inflammation, oxidative stress, neuroplasticity, mitochondrial function, and the gut microbiome that are affected by dietary intake may affect the likelihood of developing depression.^[5]^ In addition to the direct effects on macro- and micronutrients in the brain, dietary habits have a significant impact on the composition and functioning of the human gut microbiota and play a causal role in the regulation of the gut microbiota, thereby affecting mood and cognitive function.^[6]^ Diet regulates various pathophysiological processes in the human body and affects the biosynthesis of neurotransmitters and neuropeptides in the brain.^[7]^ Therefore, understanding the causal links between dietary habits and depression may be crucial for preventing depression.^[8]^

A randomized controlled trial using Bayes confirmed that a Mediterranean diet improved depressive symptoms in young men.^[9]^ Adherence to a healthy diet, mainly the Mediterranean diet, and avoidance of a pro-inflammatory diet were associated with a reduced risk of depression or depressive symptoms.^[10]^ Lu et al showed a linear dose-response relationship between the EAT-Lancet diet and a reduced risk of depression, anxiety, and their co-occurrence.^[11]^ Although the above evidence suggests that a healthy diet can help prevent the occurrence of MDD and alleviate depressive symptoms, the effects of different types of foods on MDD have not been consistently reported. Observational studies have shown that people who abstain from meat have a higher risk of depression.^[12]^ In addition, drinking alcohol, tea, and coffee may also have an impact on depression, but the results are mixed.^[13]^ Although these studies controlled for various confounding factors, residual confounding factors, such as small sample size, flawed study design, and beyond the scope of existing research, are still at risk of bias.^[14]^

We selected 16 dietary habits covering 8 major food categories based on their common consumption patterns and prior observational evidence linking them to mental health outcomes. These dietary factors were chosen due to their frequent inclusion in prior observational studies on depression and their hypothesized mechanistic links to inflammation, neurotransmitter synthesis, and gut microbiota modulation.MR was chosen as it minimizes residual confounding and reverse causality, which are common limitations in observational studies and difficult to address in randomized controlled trials due to long-term dietary adherence challenges and ethical constraints in assigning potentially harmful diets. This study used MR to investigate the relationships between different dietary habits and MDD, and whether these dietary habits constitute protective or risk factors for MDD. MR using genetic tools to reduce potential confounding bias may be an appropriate study design to evaluate the effects of diet on disease or health outcomes.^[15]^

## 2. Materials and methods

We exclusively utilize publicly available genome-wide association studies (GWAS) summary statistics, eliminating the need for additional ethical approvals or informed consent.

### 2.1. Study design and MR hypotheses

Data were extracted from published meta-analyses of GWAS, the UK Biobank, and a pooled dataset from the Psychiatric Genomics Consortium (PGC).

**Figure 1.**
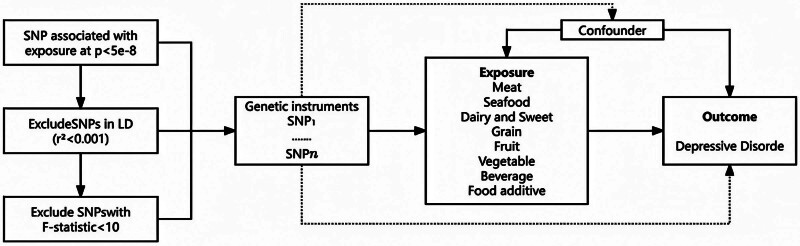
Schematic diagram of the 3 hypotheses of MR research. MR = Mendelian randomization.

Single nucleotide polymorphisms (SNPs) were used as surrogate markers for phenotypes and genetic instrumental variables in the two-sample MR studies. To investigate the relationships between dietary habits and depression, we applied 3 assumptions to genetic variants: the association hypothesis, that is, the SNP is strongly associated with exposure; the independence hypothesis, that is, the SNP is not affected by confounding factors on the exposure-outcome path; and the exclusivity hypothesis, that is, the SNP affects the outcome only through exposure but not other pathways.^[16]^ We acknowledge that the independence assumption may be violated if SNPs are associated with confounders such as socioeconomic status or physical activity. To address this, we used publicly available GWAS data that were already adjusted for principal components of ancestry and, where available, basic demographic factors. Additionally, we conducted sensitivity analyses (MR-Egger and MR-PRESSO) to detect and correct for horizontal pleiotropy, which may reflect unmeasured confounding. The study design is shown in Figure 1.

### 2.2. Data source

#### 2.2.1. Exposure data

In this study, we specifically focused on 16 different dietary habits as exposure factors, covering 8 major categories: processed meat, seafood, dairy products and desserts, cereals, fruits, vegetables, beverages, and salt in food additives. These data were extracted from the GWAS conducted by the UK Biobank, a large-scale prospective study involving approximately 500,000 participants aged 38 to 73 years, which provides genetic and phenotypic information, comprehensive descriptions of the research design, participants, and quality control methods that have been published. Summary data on GWAS dietary habits were collected from the Open GWAS project of the MRC Integrative Epidemiology Unit at the University of Bristol, which was organized by Sudlow et al and maintained by the MRC Integrative Epidemiology Unit at the University of Bristol.^[17]^

#### 2.2.2. Outcome data

We obtained GWAS summary data on depression from the PGC, as provided by the UK Biobank. The UK Biobank dietary GWAS and the PGC depression GWAS are largely independent cohorts with minimal sample overlap. Where overlap exists, its impact is expected to be minimal given the large sample sizes and the use of genetic instruments derived from independent GWAS. These 2 datasets are largely independent, with minimal sample overlap. This dataset included at least 2000 cases of depression and 482,598 controls, and all of European ancestry. A total of 9,587,836 SNPs was identified for our analysis.^[18]^ Detailed information is listed in Table 1.

**Table 1:** The GWAS datasets used in this MR study.

ID	Food type	Food intake	Sample size	Number of SNPs
ukb-b-6324	Meat	Processed meat	461,981	9,851,867
ukb-b-2862	Meat	Beef	461,053	9,851,867
ukb-b-5640	Meat	Pork	460,162	9,851,867
ukb-b-14719	Meat	Lamb mutton	460,006	9,851,867
ukb-b-17627	Seafood	Non oily fish	460,880	9,851,867
ukb-b-2209	Seafood	Oily fish	460,443	9,851,867
ukb-b-11348	Dairy and sweet	Bread	452,236	9,851,867
ukb-b-1489	Dairy and sweet	Cheese	451,486	9,851,867
ukb-b-15926	Grain	Cereal	441,640	9,851,867
ukb-b-3881	Fruit	Fresh fruit	446,462	9,851,867
ukb-b-16576	Fruit	Dried fruit	421,764	9,851,867
ukb-b-8089	Vegetable	Cooked vegetable	448,651	9,851,867
ukb-b-1996	Vegetable	Salad raw vegetable	435,435	9,851,867
ukb-b-6066	Beverage	Tea	447,485	9,851,867
ukb-b-5779	Beverage	Alcohol	462,346	9,851,867
ukb-b-8121	Food additive	Salt added to food	462,630	9,851,867

GWAS = genome-wide association studies, MR = Mendelian randomization, SNPs = single nucleotide polymorphisms.

### 2.3. Identification of instrumental variables

We screened the GWAS data based on the assumptions of MR research. The selection criteria for relevant SNPs included: Preliminary screening of SNPs that are closely related to 16 dietary habits with genome-wide significance (*P* < 5 × 10^−8^). We used a genome-wide significance threshold of *P* < 5 × 10^−8^ for SNP selection, which is the standard threshold in GWAS to minimize false-positive associations due to multiple testing. This stringent threshold ensures that the selected SNPs are robustly associated with the exposure, satisfying the first MR assumption; Use PLINK to cluster the identified SNPs and perform linkage disequilibrium aggregation of the identified SNPs within a 10,000 kb window with a strict cutoff of *R*^2^ = 0.001, referring to European samples from the 1000 Genomes Project.^[19]^ If there was an linkage disequilibrium effect between SNPs, the SNPs with the lowest *P* values were retained; To address the potential bias of weak instruments, the *F* statistic was calculated according to the established method, and SNPs with *F* statistics lower than 10 were excluded.^[20]^

### 2.4. Mendelian randomization study and sensitivity analysis

In this MR study, we used the inverse variance weighting (IVW) method to explore the causal relationship between dietary habits and depression. To ensure the robustness of the results, we performed sensitivity analyses using weighted median (IVW) and Mendelian randomization-Egger regression based on Egger regression (MR-Egger).^[21]^

For the sensitivity analyses, we calculated Cochran *Q* statistic using both IVW and MR-Egger regressions. A *P* value > .05 indicates no significant heterogeneity. Additionally, we employed the leave-one-out method, systematically excluding each included SNP individually, and generated forest plots. A *P* value > .05 after excluding an SNP suggests that the SNP did not significantly affect the results. To assess pleiotropy, we used both the intercept term of the MR-Egger regression and the Mendelian randomization pleiotropy residual sum and outlier (MR-PRESSO) test for the included SNPs. The MR-PRESSO global test was first performed to detect horizontal pleiotropy. If significant (*P* < .05), outlier SNPs were identified and removed, and the causal estimate was re-calculated using the outlier-corrected IVW method. In the MR-Egger regression, an intercept trending towards zero indicates the absence of horizontal pleiotropy. In this MR analysis, the odds ratio (OR) was used as the effect value and a 95% confidence interval (CI) was applied. Statistical significance was set at *P* < .05. The R 4.0.3 software, along with the two-sample-MR 0.5.11 and MR-PRESSO packages, were used for data processing and visualization.^[22]^

## 3. Results

### 3.1. The impact of dietary habits on depression

We assessed the impact of various dietary exposures on the results, and the *F* statistic for the identified SNPs exceeded the empirical threshold of 10, indicating that the calculated results were not susceptible to bias caused by a weak IV. The relationship between eating habits and depression was determined based on the values obtained using the IVW method, as shown in Table 2.

**Table 2:** Causal relationships between eating habits and depression.

Trait	nsnp	*P*-val	β	SE	OR
Processed meat intake	23	.3131	−0.0106	0.0105	0.9895
Beef intake	**15** *	**.0003** *	−0.0624*	**0.0174** *	**0.9395** *
Pork intake	14	.1525	−0.0237	0.0166	0.9766
Non oily fish intake	11	.1223	0.021	0.0136	1.0213
Oily fish intake	**60** *	**.0252** *	**0.0575** *	**0.025** *	**1.0592** *
Bread intake	**30** *	**.042** *	−0.0712*	**0.0334** *	**0.9313** *
Cheese intake	**63** *	**.0468** *	−0.0414*	**0.0221** *	**0.9594** *
Cereal intake	39	.2599	−0.0085	0.0076	0.9915
Fresh fruit intake	53	.8727	−0.0018	0.0113	0.9982
Dried fruit intake	41	.1753	0.0134	0.0099	1.0135
Cooked vegetable intake	17	.6826	0.0069	0.0168	1.0069
Salad raw vegetable intake	19	.4244	0.0141	0.0176	1.0142
Tea intake	39	.0642	0.011	0.006	1.0111
Alcohol intake frequency	**96** *	**.0341** *	**0.0085** *	**0.004** *	**1.0085** *
Salt added to food	**102** *	**.0001** *	**0.018** *	**0.0048** *	**1.0182** *

nsnp = number of single nucleotide polymorphisms, OR = odds ratio, *P*-val = probability-value, SE = standard error, β = beta.

* The bold black font signifies statistical significance.

Our results showed that genetically predicted to be associated with 5 dietary habits, including Salt added to food (OR: 1.0182, 95% CI: 0.0087–0.0273, *P* = .0001), Beef intake (OR: 0.9395, 95% CI: −0.0964 to −0.0284, *P* = .0003), Oily fish intake (OR: 1.0592, 95% CI: 0.0084–0.1065, *P* = .0252), Bread intake (OR: 0.9313, 95% CI: −0.1366 to −0.0057, *P* = .0420), Alcohol intake frequency (OR: 1.0085, 95% CI: 0.0006–0.0163, *P* = .0341). Both remained significant after FDR, indicating that depression was significantly related to beef intake (*b* = −0.0624, SE = 0.173, *P* = .004) and salt intake (*b* = 0.0173, SE = 0.0392, *P* = .0001).

**Figure 2.**
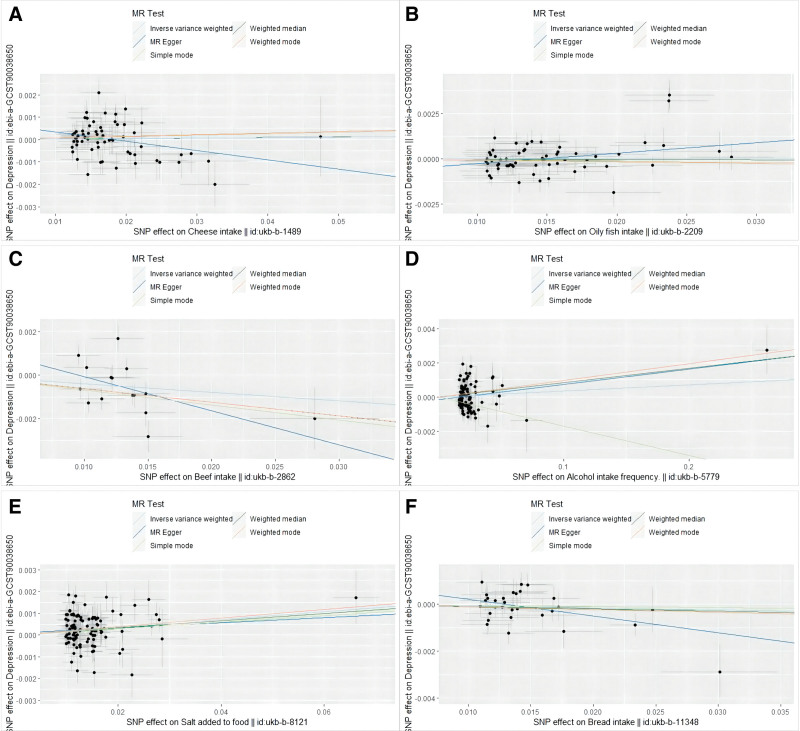
Scatter plots of the causal relationships based on the MR methods. (A) cheese intake; (B) oily fish intake; (C) beef intake; (D) alcohol intake frequency; (E) salt added to food; and (F) bread intake. MR = Mendelian randomization.

We conducted a more stringent Bonferroni follow-up, and the results supported the FDR results. The results of the WM method are consistent with those of the IVW method. Scatter plots were created to visualize the results (Fig. 2).

### 3.2. Heterogeneity and pleiotropy analysis

**Figure 3.**
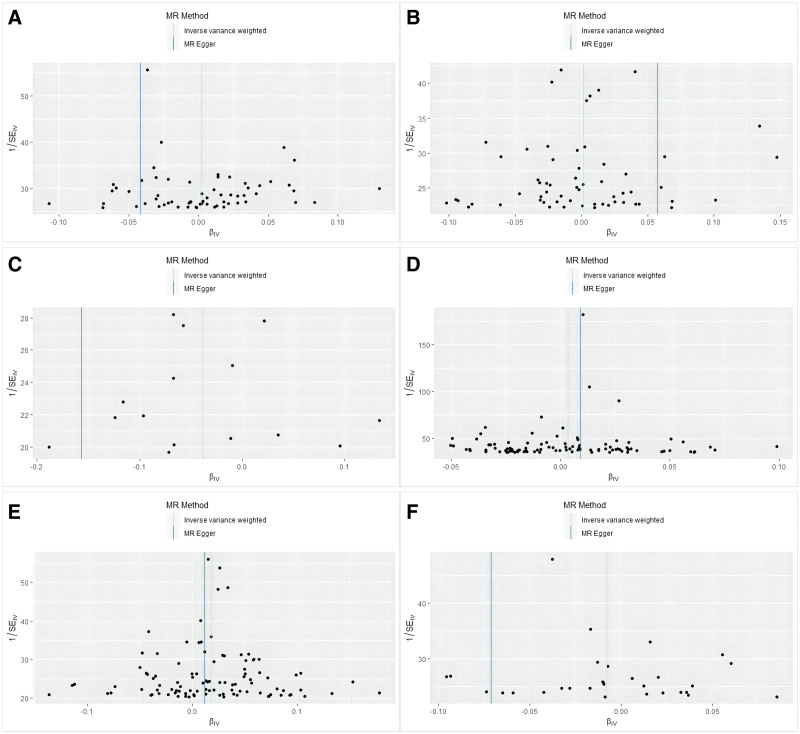
Funnel plot showing reflecting pleiotropic effects. (A) cheese intake; (B) oily fish intake; (C) beef intake; (D) alcohol intake frequency; (E) salt added to food; and (F) bread intake.

We used the IVW method in the non-parametric test (Cochran *Q* test) of related multiple samples and the MR-Egger method to detect and evaluate the heterogeneity between exposure factors and outcomes. There was no heterogeneity observed for cheese intake (*P* = .09), beef intake (*P* = .27), oily fish intake (*P* = .39), alcohol intake frequency (*P* = .74), or bread intake (*P* = .17), but the salt added to food (*P* = .04) showed certain heterogeneity. However, despite these heterogeneous properties, it did not significantly impair the structural reliability of the IVW method. Through the MR-Egger intercept test, the pleiotropy of the data was detected, and the robustness of the results was assessed. The results showed that cheese intake (*P* = .05), beef intake (*P* = .23), oily fish intake (*P* = .19), alcohol intake frequency (*P* = .34), bread intake (*P* = .06), and salt added to food (*P* = .66) were not associated with pleiotropy. MR-PRESSO identified and removed outlier SNPs for traits showing heterogeneity (e.g., salt intake). The corrected estimates remained consistent in direction and significance, supporting the robustness of our primary findings. As shown in Figure 3, there was no need to re-select the variable tool or draw a funnel chart to display the results.

### 3.3. Leave-one-out sensitivity analysis

**Figure 4.**
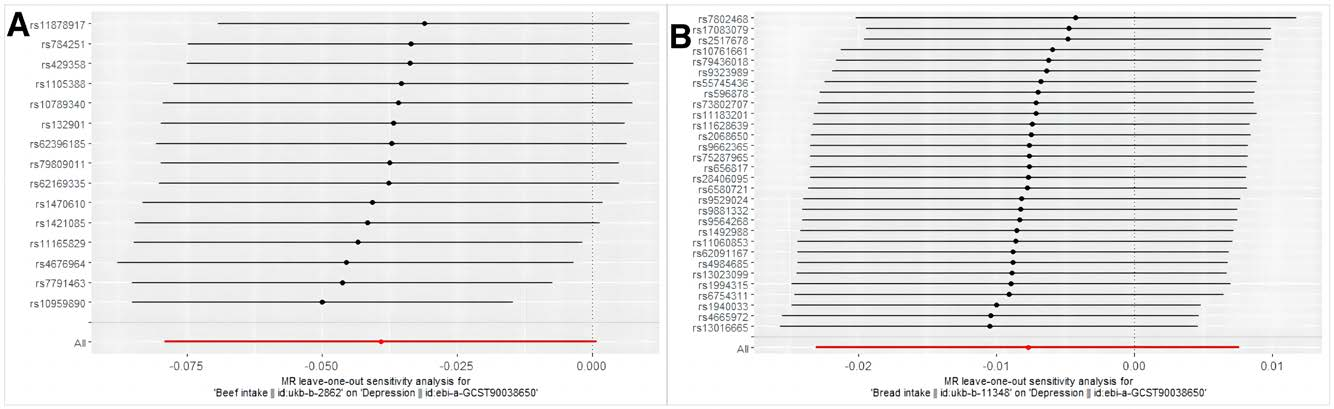
Leave-one-out graph showing causal relationships. (A) Beef intake and (B) Bread intake.

We performed leave-one-out analysis for sensitivity, excluding one significant SNP at a time. Although the MR leave-one-out estimates were not statistically significant, the effects of different dietary habits on depression were similar to the main results of MR. As shown in Figure 4, removing the SNPs individually from the 15 SNPs in beef intake and the 30 SNPs in bread intake had little impact on the overall situation.

## 4. Discussion

In this two-sample MR, we systematically studied the causal relationship between 16 dietary habits and depression. We found that genetically predicted higher salt intake, alcohol intake frequency, and oily fish intake were associated with an increased odds of depression, whereas genetically predicted higher intake of beef, bread, and cheese were associated with a reduced odds of depression. After performing MR-PRESSO to correct horizontal pleiotropy by outlier removal, the results remained consistent. We did not find a causal relationship between other dietary habits and depression. While the observed ORs (e.g., 1.018 for salt) are modest, they are consistent with the polygenic nature of depression and the multifactorial influence of diet. Such effects, though small at the individual level, may hold public health relevance at the population level given the widespread exposure to these dietary factors.

We found that beef consumption had a genetically predicted association with lower risk of depression, but the consumption of other meats such as pork, poultry, and other processed meats had no such effect. A meta-analysis was conducted by Zhang et al^[23]^ concluded that meat intake was not associated with depression. A possible reason for this may be that the meat in their research included different types. The meat in this study was divided into 4 different types that were used to minimize residual confounding factors, and genetic variation was used as an instrumental variable for meat intake, thereby more accurately elucidating the causal relationship between beef intake and depression. Beef is rich in protein as well as a large amount of zinc, iron, and B vitamins, which may be crucial for maintaining muscle mass. Lower muscle mass has been associated with a higher incidence of depression.^[24,25]^

Previous studies have suggested that increasing whole-grain intake may prevent the occurrence of depression,^[26]^ while others have suggested that grain intake has no protective effect on depression in the elderly population.^[27]^ Our study found that bread intake has a genetically predicted protective association, and a potential explanation for this effect may be that grains can regulate the intestinal microbial flora and then affect the occurrence of depression through the mediation of the flora.^[28]^

Our study found that genetically predicted oily fish intake was associated with higher depression risk, while a meta-analysis of more than 10 prospective cohort studies found that increasing one serving of fish per week had no significant effect on depression.^[29]^ Considering that oily fish may have fewer unsaturated fatty acids than non-oily fish, including omega-3, it is possible that adequate amounts of docosahexaenoic acid and eicosapentaenoic acid can combat the occurrence of depression.^[30]^ The counterintuitive association between oily fish and depression may reflect residual confounding (e.g., preparation methods, environmental contaminants) or the specific fatty acid composition in the studied population. Alternatively, it may indicate a non-linear relationship where moderate intake is beneficial but high intake is not.

A meta-analysis showed that patients with alcohol use disorder are prone to suffer depressive symptoms, while in other cases, daily drinking was not associated with the risk of depression.^[31]^ In contrast, another study found that moderate drinking may reduce the risk of depressive symptoms.^[22]^ This study found that alcohol consumption is indeed genetically associated with higher depression risk. Given the addiction risk of alcohol and its competition with antidepressants, as well as the fact that alcohol can cause decreased levels of consciousness and impaired cognitive function, depressed patients and their high-risk groups should abstain from alcohol.

This study also found a relationship between cheese consumption and depression. Since cheese can provide a lot of nutrients such as sugar and fat, studies have shown that high intake of calories and fat could reduce the level of serotonin transporter in the hypothalamus, and also increase immune inflammatory events and mitochondrial damage.^[24]^ It can induce neuroadaptive changes in the brain’s reward system and the effects of metabolic byproducts, oxidative stress, and inflammation, thereby potentially influencing the occurrence of depression.^[32]^

Previous studies have shown that a diet of specific nutrients, foods (fish, nuts, vegetables, coffee), or dietary supplements is one of the most promising ways to intervene in depression^[33]^; however, we did not find a link between other eating habits and depression. Based on the current evidence, we believe that excessive intake of salt, alcohol, and oily fish should be avoided; however, due to the heterogeneity and pleiotropic bias in MR analysis, as well as the assumption of SNPs as variable tools, our results should be interpreted with caution.

Several limitations should be acknowledged. First, the effect sizes (ORs) observed, though statistically significant, are modest (e.g., 1.018 for salt). While such effects may have public health relevance at the population level, their individual clinical significance may be limited. Second, our study participants were of European ancestry, limiting the generalizability of findings to other ethnic groups. Third, dietary exposures were self-reported, which may be subject to measurement error and misclassification. Fourth, although we employed multiple sensitivity analyses, the possibility of residual horizontal pleiotropy cannot be entirely ruled out. Finally, MR estimates represent lifelong genetic predisposition and may not directly equate to the effects of short-term dietary changes.

## 5. Conclusion

This study identified the genetically predicted associations between specific dietary habits and depression. High be associated with an increased risk of salt, alcohol, and oily fish may increase the risk of depression, while the intake of milk, bread, and cheese may be protective factors for depression. The intake of beef, rich in vitamins, minerals, and proteins, as well as foods like bread and cheese, may influence depression risk through pathways involving gut microbiota and inflammatory responses. However, it should be noted that the relationships between diet and mental health is complex and involves multiple factors and mechanisms. Therefore, our results should be interpreted with caution, and further studies are needed to validate these results.

## Author contributions

**Conceptualization:** Jianmin Liu, Juan Jia, Hai Yuan, Derong Kong, Wenfeng Li.

**Data curation:** Meng Li, Jianmin Liu, Wenfeng Li.

**Formal analysis:** Meng Li, Jianmin Liu.

**Funding acquisition:** Kun Wang.

**Investigation:** Meng Li, Kun Wang.

**Methodology:** Meng Li, Derong Kong.

**Project administration:** Juan Jia, Hai Yuan.

**Resources:** Kun Wang, Juan Jia, Hai Yuan.

**Software:** Kun Wang, Hai Yuan, Wenfeng Li.

**Validation:** Juan Jia, Derong Kong, Wenfeng Li.

**Visualization:** Wenfeng Li.

**Writing – original draft:** Meng Li, Wenfeng Li.

**Writing – review & editing:** Meng Li, Kun Wang, Wenfeng Li.
